# The Royal Army Medical Corps

**Published:** 1902-05-17

**Authors:** 


### The Hospital

A Journal of

The Medical Sciences and Hospital Administration.

Vol. XXXII.—No. 816. Edited by Sir Henry Burdett, K.C.B., and Solomon C. Smith, M.D., M.R.C.P. [May 17, 1902.

### The Royal Army Medical Corps.

The War Office has now given practical effect to the recommendations of Mr. Brodrick's Committee on the conditions of pay and service in the Royal Army Medical Corps, and we think it will be admitted, by the great majority of competent critics, that the regulations issued last week are not only a great improvement upon any which have preceded them, but that they will suffice, if cordially accepted and scrupulously acted upon, to remove the greater part of the grievances of which the medical officers of the Army have hitherto had to complain, and to justify a hope that the corps will for the future offer considerable attractions to many of the most promising of the annual additions to the ranks of the medical profession. There is, to begin with, a definite increase in the pay of all grades of the officers in the corps; an increase not only salutary and convenient to the recipients, and not only rendering the pecuniary prospects of the average medical officer approximately equal to, even if not somewhat better than, those of the average medical civilian, but also serving to indicate, in an unmistakable way, the increased estimation in which the service is for the future to be held by the State. In Kinglake's "History of the Invasion of the Crimea" there is a striking passage forming part of the description of the several causes which combined to bring about the terrible disasters of the winter of 1854-5, and explaining the manner in which, in departments not animated by political ambition, public servants are prone to imagine that they can measure the official authority really vested in one of their number by seeing how Government labels him in the figures denoting his salary. The economic reformers of the earlier part of the century had, the author tells us, left standing the emptiest pomps and vanities of their country, and applied their nipping parsimony to strictly useful institutions, including that medical service upon which, in the times then to come, the health and the lives of our sick and wounded soldiery would have to depend. The service became depressed. State parsimony, it is true, did not blunt the professional skill, did not tire out the generous devotion of our army surgeons, but it weakened their self-confidence and authority; and to weaken their self-confidence and authority was to deprive them of that power of bold innovation which is hardly less needful than gunpowder at the beginning of a war. The preparations made and recommended by the then Director-General were in their nature admirable and sufficient; but, by reason of his inadequate authority and comparatively subordinate position, they were but imperfectly carried into effect. The definite recognition of the claims of the medical service to better pay cannot fail to exert a great, although indirect, influence upon the power which it will possess of securing that its recommendations are treated with the consideration which they will deserve. A good deal has been made, by some of Mr. Brodrick's critics, of the number of examinations to which officers of the corps will be called upon to submit during their ascent of the ladder of promotion; but the chief basis of such criticism has been furnished by a belief that the subjects of these examinations would be those in which the officers in question had passed at the commencement of their careers. It turns out that the examinations will either have reference to the peculiar duties of a medical as distinguished from those of a civilian doctor, or that they will be on special subjects in which extra pay will be attainable as a reward of proficiency. Combatant officers are required to pass examinations as conditions of advancement to grades in which the subjects of examination will form parts of their daily work, and in which ignorance of these subjects might be disastrous to the men under their command, or to the operations in which these men were to be engaged. It may be said that other tests are preferable to those afforded by examination, and that the work already done, and officially reported upon by superiors, should be sufficient to indicate the place which any officer is entitled to hold in the service; but there are many practical difficulties in the way of translating this pious opinion into satisfactory and orderly practice. The personal opinions of superiors are apt to be attributed to favouritism; and, due weight being given to the necessary imperfection of all human methods, we are inclined to think that the method of selection by examination, or at least of the exclusion of incompetence by examination, is, on the whole, the best that the wit of man has so far devised. Examinations would doubtless be utterly repugnant to officers, if such there were, who desired to devote the time not occupied in the performance of hospital duties to bridge or to ping-pong ; but we are convinced that no man who had really devoted thought and time to a study of the requirements of his calling, and who was anxious to understand them thoroughly and to fulfil them efficiently, would have the smallest cause to shrink from any examination by which this anxiety and the fruits of it could be tested and displayed.

